# Nitro­furan­toin methanol monosolvate

**DOI:** 10.1107/S1600536811003679

**Published:** 2011-02-02

**Authors:** Venu R. Vangala, Pui Shan Chow, Reginald B. H. Tan

**Affiliations:** a Institute of Chemical and Engineering Sciences, A*STAR (Agency for Science, Technology and Research), 1 Pesek Road, Jurong Island, Singapore 627833; b Department of Chemical & Biomolecular Engineering, National University of Singapore, 4 Engineering Drive 4, Singapore 117576

## Abstract

The anti­biotic nitro­furan­toin {systematic name: (*E*)-1-[(5-nitro-2-fur­yl)methyl­idene­amino]­imidazolidine-2,4-dione} crys­tallizes as a methanol monosolvate, C_8_H_6_N_4_O_5_·CH_4_O. The nitro­furan­toin mol­ecule adopts a nearly planar conformation (r.m.s. deviation = 0.0344 Å). Hydrogen bonds involve the co-operative N—H⋯O—H⋯O heterosynthons between the cyclic imide of nitro­furan­toin and methanol O—H groups. There are also C—H⋯O hydrogen bonds involving the nitro­furan­toin mol­ecules which support the key hydrogen-bonding synthon. The overall crystal packing is further assisted by weak C—H⋯O inter­actions, giving a herringbone pattern.

## Related literature

For polymorphism and pseudopolymorphs, see: Bernstein (2002 ▶); Byrn *et al.* (1999 ▶); Aitipamula *et al.* (2010 ▶). For nitro­furan­toin hydrate and anhydrate crystal structures, see: Otsuka *et al.* (1991 ▶); Pienaar *et al.* (1993*a* ▶,*b* ▶); Bertolasi *et al.* (1993) ▶ and for nitro­furan­toin pseudopolymorphs, see: Caira *et al.* (1996 ▶); Tutughamiarso *et al.* (2011 ▶). For a 1:1 co-crystal involving nitro­furan­toin and 4-hy­droxy­benzoic acid, see: Vangala *et al.* (2011 ▶). For hydrogen bonding, see: Desiraju & Steiner (1999 ▶); Desiraju (2002 ▶, 2007 ▶). 
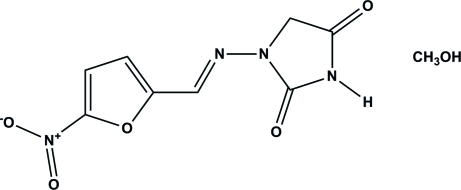


## Experimental

### 

#### Crystal data

C_8_H_6_N_4_O_5_·CH_4_O
*M* *_r_* = 270.21
Monoclinic, 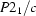

*a* = 6.4084 (13) Å
*b* = 6.5941 (13) Å
*c* = 26.705 (5) Å
β = 91.70 (3)°
*V* = 1128.0 (4) Å^3^
*Z* = 4
Mo *K*α radiation
μ = 0.14 mm^−1^
*T* = 110 K
0.13 × 0.11 × 0.11 mm

#### Data collection

Rigaku Saturn 70 CCD area-deterctor diffractometer
Absorption correction: multi-scan (*CrystalClear*; Rigaku, 2008 ▶) *T*_min_ = 0.983, *T*_max_ = 0.985
17441 measured reflections
3299 independent reflections
2849 reflections with *I* > 2σ(*I*)
*R*_int_ = 0.048

#### Refinement

*R*[*F* ^2^ > 2σ(*F* ^2^)] = 0.071
*wR*(*F* ^2^) = 0.129
*S* = 1.24
3299 reflections
212 parameters
All H-atom parameters refined
Δρ_max_ = 0.24 e Å^−3^
Δρ_min_ = −0.26 e Å^−3^

### 

Data collection: *CrystalClear* (Rigaku, 2008) ▶; cell refinement: *CrystalClear* ▶; data reduction: *CrystalClear* ▶; program(s) used to solve structure: *SHELXS97* (Sheldrick, 2008 ▶); program(s) used to refine structure: *SHELXL97* (Sheldrick, 2008 ▶); molecular graphics: *X-SEED* (Barbour, 2001 ▶); software used to prepare material for publication: *SHELXTL* (Sheldrick, 2008 ▶) and *PLATON* (Spek, 2009 ▶).

## Supplementary Material

Crystal structure: contains datablocks global, I. DOI: 10.1107/S1600536811003679/ng5112sup1.cif

Structure factors: contains datablocks I. DOI: 10.1107/S1600536811003679/ng5112Isup2.hkl

Additional supplementary materials:  crystallographic information; 3D view; checkCIF report

## Figures and Tables

**Table 1:** Hydrogen-bond geometry (Å, °)

*D*—H⋯*A*	*D*—H	H⋯*A*	*D*⋯*A*	*D*—H⋯*A*
N4—H4⋯O6	0.88 (3)	1.88 (3)	2.755 (2)	170 (3)
O6—H6⋯O5^i^	0.86 (3)	1.93 (3)	2.787 (2)	172 (3)
C5—H5⋯O4^ii^	0.95 (2)	2.22 (2)	3.155 (2)	169.2 (18)
C3—H3⋯O3^ii^	0.94 (2)	2.42 (2)	3.176 (3)	138 (2)

Symmetry codes: (i) 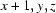; (ii) 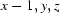.

**Fig. 1.**
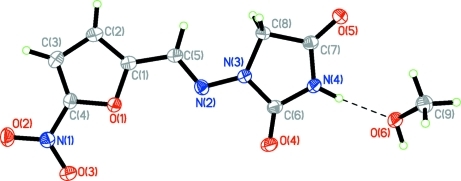
A perspective view showing the molecular structures of nitrofurantoin and methanol, with atom labels and 50% probability displacement ellipsoids for non-H atoms. The dashed line shows the N—H···O hydrogen bond.

**Fig. 2.**
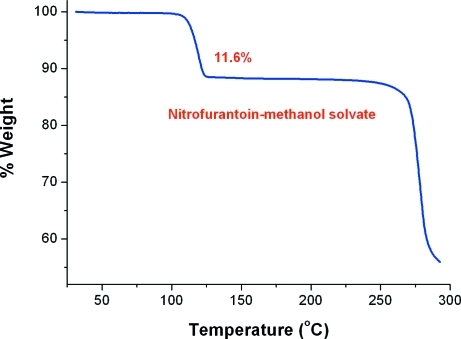
TGA traces showed that there was a weight loss of 11.6% (w/w), which can be attributed to a methanol solvate.

**Fig. 3.**
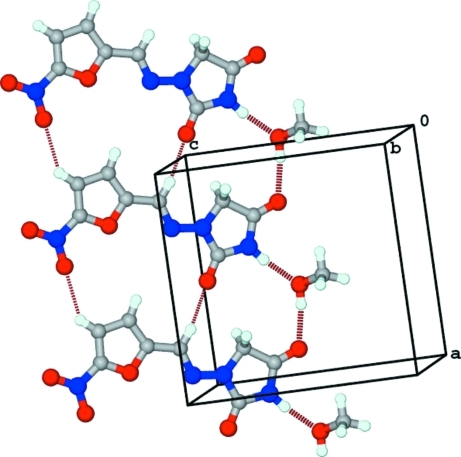
A partial packing diagram of the title pseudopolymorph is viewed down b-axis, showing formation of the N—H···O—H···O synthon along a-axis. Notice the strong C—H···O hydrogen bonds within nitrofurantoin molecules to support the solid-state structure.

**Fig. 4.**
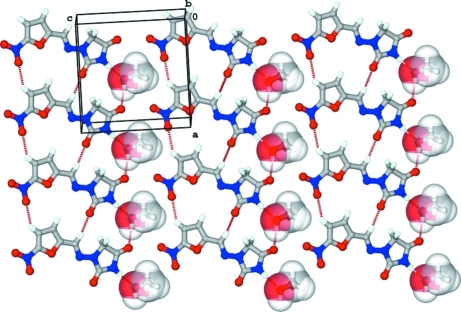
A partial packing diagram of the title pseudopolymorph is viewed down b-axis, showing ribbons running along a-axis. Notice the methanol monosolvate showed in space filled style.

**Fig. 5.**
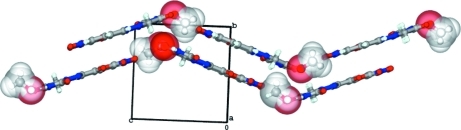
Crystal packing of the title pseudopolymorph is viewed down the a-axis, showing a herringbone pattern. Notice also the methanol monosolvate showed in space filled style.

## supplementary crystallographic information

### Comment

Polymorphism is an ability of a molecule to exist in two or more crystal structures. Incorporation of solvent molecules into the crystalline lattice are routinely referred to as solvates, inclusion complexes and/or pseudopolymorphs (Bernstein, 2002; Byrn *et al.*, 1999; Aitipamula *et al.*, 2010). A full characterization of various crystal forms of an active pharmaceutical ingredient (API) may reveal desired physical form. Thus, it is relevant to pharmaceutical industry. Nitrofurantoin {(*E*)-1-[(5-nitro-2-furyl)methylideneamino]imidazoldine-2,4-dione} is an antibacterial agent used in the treatment of genitourinary tract infections. It exists in both anhydrous (α- and β-) and hydrate forms (Forms I and II) (Pienaar *et al.*, 1993*a*, 1993*b*; Bertolasi *et al.*, 1993), and literature findings show that nitrofurantoin has poor physical properties (Otsuka *et al.*, 1991; Caira *et al.*, 1996). We have recently reported a 1:1 co-crystal involving nitrofurantoin and 4-hydroxybenzoic acid and shown that co-crystal displayed superior physicochemical and photo-stability to that of nitrofurantoin. However, co-crystallization attempt of nitrofurantoin with fumaric acid in methanol instead yielded the title pseudopolymorph, nitrofurantoin methanol monosolvate. It was reported that this API is known to form inclusion complexes with dimethylformamide, dimethyl sulfoxide and dimethylacetamide (Caira *et al.*, 1996; Tutughamiarso *et al.*, 2011). Herein we report the structural features of a 1:1 pseudopolymorph involving nitrofurantoin and methanol (Fig. 1).

Thermogravimetric analysis (TGA traces) of the title compound is shown in Fig. 2. The measured weight loss (11.6% *w*/*w*) in the temperature range of 110–140 °C is in agreement with the stoichiometric weight content for a methanol monosolvate (11.8% *w*/*w*).

Single crystal X-ray diffraction analysis reveals that the crystal structure contains one molecule each of nitrofurantoin and methanol in the asymmetric unit (Fig. 1). It has crystallized in the monoclinic crystal system with *P*2_1_/*c* space group. In the structure, nitrofurantoin and methanol molecules were essentially held together by a primary co-operative synthon of N—H···O—H···O [*D*/Å, *θ*/°: 2.755 (2), 170 (3); 2.787 (2), 172 (3)] between amide N4—H4, methanolic O6—H6 and imide O5 along the *a*-axis (Fig. 3). In addition, there are significant C—H···O hydrogen bonds (Desiraju & Steiner 1999; Desiraju 2002; Desiraju 2007) within nitrofurantoin molecules, which lead to ribbons running along *a*-axis and support the key hydrogen bonding synthon (Fig. 4). It has structural reminiscences with anhydrous nitrofurantoin (β-form), where the packing is stabilized by imide catemer of N—H···O interactions (Pienaar *et al.*, 1993*b*; Bertolasi *et al.*, 1993). Here, however, it is replaced with N—H···O—H···O catemer type of heterosynthon by retaining a two fold screw axis. Recently, we have noted an identical synthon in a 1:1 co-crystal involving nitrofurantoin and phenolic co-former (4-hydroxybenzoic acid) (Vangala *et al.*, 2011). Hence, supramolecularly methnolic O–H interacted similar to what phenolic O–H is able to do in the reported co-crystal. The overall crystal packing of the title pseudopolymorph is further assisted by weak C—H···O interactions to give a herringbone type of pattern (Fig. 5). Further analysis showed that this herringbone pattern was comparable to that of a crystal structure of nitrofurantoin dimethyl sulfoxide monosolvate (Tutughamiarso *et al.*, 2011).

### Experimental

The title psuedopolymorph was obtained by evaporative crystallization during attempts to co-crystallize a commercially available (purchased from Aldrich) nitrofurantoin (β-form, 119 mg, 0.5 mmol) with fumaric acid (58 mg, 0.5 mmol) in methanol (25 ml) at ambient conditions. The yellow needle shaped crystals suitable for single-crystal X-ray diffraction were obtained in three days.

### Refinement

All H atoms bonded to C, N, O atoms were located in a difference map and allowed to ride on their parent atoms in the refinement cycles.

### Crystal data

**Table:**

C_8_H_6_N_4_O_5_·CH_4_O	*D*_x_ = 1.591 Mg m^−^^3^
*M**_r_* = 270.21	Melting point: 547 K
Monoclinic, *P*2_1_/*c*	Mo *K*α radiation, λ = 0.71073 Å
*a* = 6.4084 (13) Å	Cell parameters from 2901 reflections
*b* = 6.5941 (13) Å	θ = 1.5–31.2°
*c* = 26.705 (5) Å	µ = 0.14 mm^−^^1^
β = 91.70 (3)°	*T* = 110 K
*V* = 1128.0 (4) Å^3^	Block, yellow
*Z* = 4	0.13 × 0.11 × 0.11 mm
*F*(000) = 560	

### Data collection

**Table:**

Rigaku Saturn 70 CCD area-deterctor diffractometer	3299 independent reflections
Radiation source: fine-focus sealed tube	2849 reflections with *I* > 2σ(*I*)
graphite	*R*_int_ = 0.048
ω scans	θ_max_ = 31.2°, θ_min_ = 3.2°
Absorption correction: multi-scan (*CrystalClear*; Rigaku, 2008)	*h* = −6→9
*T*_min_ = 0.983, *T*_max_ = 0.985	*k* = −8→9
17441 measured reflections	*l* = −37→37

### Refinement

**Table:**

Refinement on *F*^2^	Primary atom site location: structure-invariant direct methods
Least-squares matrix: full	Secondary atom site location: difference Fourier map
*R*[*F*^2^ > 2σ(*F*^2^)] = 0.071	Hydrogen site location: inferred from neighbouring sites
*wR*(*F*^2^) = 0.129	All H-atom parameters refined
*S* = 1.24	*w* = 1/[σ^2^(*F*_o_^2^) + (0.033*P*)^2^ + 0.7077*P*] where *P* = (*F*_o_^2^ + 2*F*_c_^2^)/3
3299 reflections	(Δ/σ)_max_ < 0.001
212 parameters	Δρ_max_ = 0.24 e Å^−^^3^
0 restraints	Δρ_min_ = −0.26 e Å^−^^3^

### Special details

**Table:**

Geometry. All e.s.d.'s (except the e.s.d. in the dihedral angle between two l.s. planes) are estimated using the full covariance matrix. The cell e.s.d.'s are taken into account individually in the estimation of e.s.d.'s in distances, angles and torsion angles; correlations between e.s.d.'s in cell parameters are only used when they are defined by crystal symmetry. An approximate (isotropic) treatment of cell e.s.d.'s is used for estimating e.s.d.'s involving l.s. planes.
Refinement. Refinement of *F*^2^ against ALL reflections. The weighted *R*-factor *wR* and goodness of fit *S* are based on *F*^2^, conventional *R*-factors *R* are based on *F*, with *F* set to zero for negative *F*^2^. The threshold expression of *F*^2^ > σ(*F*^2^) is used only for calculating *R*-factors(gt) *etc*. and is not relevant to the choice of reflections for refinement. *R*-factors based on *F*^2^ are statistically about twice as large as those based on *F*, and *R*- factors based on ALL data will be even larger.

### Fractional atomic coordinates and isotropic or equivalent isotropic displacement parameters (Å^2^)

**Table:**

	*x*	*y*	*z*	*U*_iso_*/*U*_eq_	
O6	0.9997 (2)	−0.1389 (2)	0.22463 (5)	0.0263 (3)	
C9	0.9409 (4)	−0.0216 (3)	0.18136 (8)	0.0278 (4)	
H9C	0.997 (4)	0.115 (4)	0.1824 (10)	0.042 (7)*	
H9B	0.990 (4)	−0.085 (4)	0.1496 (10)	0.039 (7)*	
H9A	0.786 (4)	−0.017 (4)	0.1807 (10)	0.047 (8)*	
H6	1.134 (5)	−0.139 (4)	0.2267 (10)	0.046 (8)*	
O3	0.6808 (2)	0.3602 (2)	0.58168 (5)	0.0284 (3)	
N3	0.5389 (2)	0.0501 (2)	0.36110 (6)	0.0215 (3)	
O2	0.4103 (2)	0.4243 (2)	0.62695 (5)	0.0295 (3)	
C7	0.5011 (3)	−0.0753 (3)	0.28106 (7)	0.0215 (4)	
N2	0.5159 (3)	0.1188 (2)	0.40903 (6)	0.0215 (3)	
N4	0.7053 (3)	−0.0642 (3)	0.29543 (6)	0.0221 (3)	
C4	0.3538 (3)	0.3193 (3)	0.54700 (7)	0.0217 (4)	
C8	0.3751 (3)	−0.0024 (3)	0.32458 (7)	0.0212 (4)	
C1	0.2822 (3)	0.2104 (3)	0.47230 (7)	0.0210 (4)	
C6	0.7351 (3)	0.0089 (3)	0.34419 (7)	0.0218 (4)	
C3	0.1432 (3)	0.3112 (3)	0.54369 (8)	0.0250 (4)	
C2	0.0962 (3)	0.2407 (3)	0.49475 (8)	0.0238 (4)	
C5	0.3262 (3)	0.1396 (3)	0.42254 (7)	0.0214 (4)	
H5	0.208 (3)	0.109 (3)	0.4017 (8)	0.016 (5)*	
H2	−0.034 (4)	0.218 (3)	0.4794 (9)	0.027 (6)*	
H8B	0.284 (4)	−0.114 (4)	0.3365 (9)	0.028 (6)*	
O1	0.4459 (2)	0.2587 (2)	0.50437 (5)	0.0212 (3)	
H4	0.807 (4)	−0.098 (4)	0.2753 (10)	0.039 (7)*	
H3	0.053 (4)	0.348 (4)	0.5693 (9)	0.034 (7)*	
H8A	0.292 (4)	0.116 (4)	0.3134 (9)	0.038 (7)*	
O5	0.4315 (2)	−0.1331 (2)	0.24050 (5)	0.0259 (3)	
O4	0.9013 (2)	0.0278 (2)	0.36653 (5)	0.0281 (3)	
N1	0.4910 (3)	0.3720 (2)	0.58760 (6)	0.0226 (3)	

### Atomic displacement parameters (Å^2^)

**Table:**

	*U*^11^	*U*^22^	*U*^33^	*U*^12^	*U*^13^	*U*^23^
O6	0.0204 (7)	0.0356 (8)	0.0228 (7)	0.0026 (6)	0.0015 (6)	0.0021 (6)
C9	0.0291 (11)	0.0273 (10)	0.0269 (10)	−0.0001 (8)	0.0004 (8)	0.0039 (8)
O3	0.0195 (7)	0.0364 (8)	0.0294 (7)	−0.0017 (6)	0.0000 (6)	−0.0018 (6)
N3	0.0171 (8)	0.0272 (8)	0.0200 (7)	−0.0002 (6)	0.0007 (6)	−0.0018 (6)
O2	0.0340 (8)	0.0333 (8)	0.0216 (7)	−0.0004 (6)	0.0075 (6)	−0.0031 (6)
C7	0.0192 (9)	0.0216 (9)	0.0236 (9)	0.0011 (7)	−0.0004 (7)	0.0016 (7)
N2	0.0243 (8)	0.0214 (8)	0.0186 (7)	−0.0018 (6)	0.0003 (6)	0.0005 (6)
N4	0.0176 (8)	0.0279 (8)	0.0208 (8)	0.0013 (6)	0.0019 (6)	0.0001 (6)
C4	0.0229 (9)	0.0212 (9)	0.0211 (8)	0.0006 (7)	0.0037 (7)	−0.0008 (7)
C8	0.0179 (9)	0.0240 (9)	0.0215 (9)	0.0005 (7)	−0.0014 (7)	−0.0014 (7)
C1	0.0189 (9)	0.0205 (9)	0.0236 (9)	−0.0006 (7)	−0.0003 (7)	0.0020 (7)
C6	0.0184 (9)	0.0242 (9)	0.0228 (9)	−0.0016 (7)	0.0000 (7)	0.0027 (7)
C3	0.0229 (10)	0.0254 (10)	0.0269 (10)	0.0021 (8)	0.0058 (8)	0.0014 (7)
C2	0.0187 (9)	0.0250 (9)	0.0276 (10)	0.0004 (7)	−0.0003 (8)	0.0025 (7)
C5	0.0185 (9)	0.0214 (9)	0.0241 (9)	−0.0001 (7)	−0.0012 (7)	0.0003 (7)
O1	0.0196 (7)	0.0246 (7)	0.0195 (6)	0.0005 (5)	0.0015 (5)	−0.0007 (5)
O5	0.0235 (7)	0.0324 (8)	0.0217 (7)	0.0007 (6)	−0.0016 (5)	−0.0036 (5)
O4	0.0186 (7)	0.0390 (8)	0.0267 (7)	−0.0029 (6)	−0.0004 (5)	0.0026 (6)
N1	0.0246 (9)	0.0215 (8)	0.0219 (8)	−0.0017 (6)	0.0026 (6)	0.0013 (6)

### Geometric parameters (Å, °)

**Table:**

O6—C9	1.432 (2)	N4—H4	0.88 (3)
O6—H6	0.86 (3)	C4—C3	1.351 (3)
C9—H9C	0.97 (3)	C4—O1	1.358 (2)
C9—H9B	1.00 (3)	C4—N1	1.419 (3)
C9—H9A	1.00 (3)	C8—H8B	1.00 (2)
O3—N1	1.234 (2)	C8—H8A	0.99 (3)
N3—N2	1.370 (2)	C1—C2	1.365 (3)
N3—C6	1.375 (2)	C1—O1	1.372 (2)
N3—C8	1.454 (2)	C1—C5	1.444 (3)
O2—N1	1.234 (2)	C6—O4	1.212 (2)
C7—O5	1.220 (2)	C3—C2	1.411 (3)
C7—N4	1.355 (2)	C3—H3	0.94 (2)
C7—C8	1.513 (3)	C2—H2	0.93 (2)
N2—C5	1.286 (3)	C5—H5	0.95 (2)
N4—C6	1.396 (2)		
			
C9—O6—H6	107.0 (19)	N3—C8—H8A	112.7 (15)
O6—C9—H9C	112.9 (16)	C7—C8—H8A	108.3 (15)
O6—C9—H9B	112.2 (15)	H8B—C8—H8A	111.3 (19)
H9C—C9—H9B	106 (2)	C2—C1—O1	110.68 (17)
O6—C9—H9A	105.6 (16)	C2—C1—C5	130.44 (18)
H9C—C9—H9A	110 (2)	O1—C1—C5	118.88 (16)
H9B—C9—H9A	109 (2)	O4—C6—N3	128.06 (18)
N2—N3—C6	119.80 (15)	O4—C6—N4	126.05 (18)
N2—N3—C8	127.60 (15)	N3—C6—N4	105.89 (16)
C6—N3—C8	112.43 (15)	C4—C3—C2	104.99 (17)
O5—C7—N4	126.39 (18)	C4—C3—H3	125.3 (16)
O5—C7—C8	126.25 (17)	C2—C3—H3	129.7 (16)
N4—C7—C8	107.36 (16)	C1—C2—C3	106.88 (18)
C5—N2—N3	115.25 (16)	C1—C2—H2	124.4 (15)
C7—N4—C6	112.76 (16)	C3—C2—H2	128.8 (15)
C7—N4—H4	122.4 (17)	N2—C5—C1	120.33 (17)
C6—N4—H4	124.8 (17)	N2—C5—H5	124.0 (13)
C3—C4—O1	113.05 (17)	C1—C5—H5	115.7 (13)
C3—C4—N1	130.92 (18)	C4—O1—C1	104.40 (14)
O1—C4—N1	115.98 (16)	O2—N1—O3	124.47 (17)
N3—C8—C7	101.52 (15)	O2—N1—C4	116.97 (16)
N3—C8—H8B	112.3 (14)	O3—N1—C4	118.54 (16)
C7—C8—H8B	110.1 (14)		

### Hydrogen-bond geometry (Å, °)

**Table:**

*D*—H···*A*	*D*—H	H···*A*	*D*···*A*	*D*—H···*A*
N4—H4···O6	0.88 (3)	1.88 (3)	2.755 (2)	170 (3)
O6—H6···O5^i^	0.86 (3)	1.93 (3)	2.787 (2)	172 (3)
C5—H5···O4^ii^	0.95 (2)	2.22 (2)	3.155 (2)	169.2 (18)
C3—H3···O3^ii^	0.94 (2)	2.42 (2)	3.176 (3)	138 (2)

Symmetry codes: (i) *x*+1, *y*, *z*; (ii) *x*−1, *y*, *z*.
